# Atrial fibrillation monitoring with wrist-worn photoplethysmography-based wearables: State-of-the-art review

**DOI:** 10.1016/j.cvdhj.2020.03.001

**Published:** 2020-08-26

**Authors:** Linda M. Eerikäinen, Alberto G. Bonomi, Lukas R.C. Dekker, Rik Vullings, Ronald M. Aarts

**Affiliations:** ∗ Eindhoven University of Technology, Department of Electrical Engineering, Eindhoven, The Netherlands; † Philips Research, Department of Patient Care and Measurements, Eindhoven, The Netherlands; ‡ Catharina Hospital, Department of Cardiology, Eindhoven, The Netherlands

**Keywords:** Atrial fibrillation, Photoplethysmography, Wrist-worn wearables

## Abstract

Early detection and diagnosis of atrial fibrillation (AF) is essential in order to prevent stroke and other severe health consequences. The challenges in diagnosing AF arise from its intermittent and asymptomatic nature. Wrist-worn devices that use monitoring based on photoplethysmography have been proposed recently as a possible solution because of their ability to monitor heart rate and rhythm for long periods of time at low cost. Long-term continuous monitoring with implantable devices has been shown to increase the percentage of detected AF episodes, but the additional value of wrist-worn devices has yet to be determined. In this review, we present the state of the art in AF detection with wrist-worn devices, discuss the potential of the technology and current knowledge gaps, and propose directions for future research. The state-of-the-art methods show excellent accuracy for AF detection. However, most of the studies were conducted in hospital settings, and more studies showing the accuracy of the technology for ambulatory long-term monitoring are needed. Objective comparison of results and methodologies among different studies currently is difficult due to the lack of adequate public datasets.

**Key Findings**

• Atrial fibrillation detection with wrist-worn photoplethysmography-based wearables shows overall excellent accuracy.
• Most studies have been conducted by monitoring short-term, hospitalized patients. More studies in ambulatory settings and with long-term measurements are needed in order to gain more insight into detection accuracy and measurement coverage in daily life.
• Comparison of results and methodologies among different studies is challenging. Publicly available datasets would facilitate objective comparisons and further accelerate development of algorithms for atrial fibrillation detection from photoplethysmography data.

## Introduction

Atrial fibrillation (AF) is the most common sustained cardiac arrhythmia and can lead to serious health consequences, such as stroke or heart failure.^1^ The prevalence of AF is expected to increase in the future,^2^ so solutions for timely diagnosis and treatment of AF are needed in order to prevent stroke and other adverse events caused by this arrhythmia. From a population health perspective, effective screening strategies are needed.^3^

Several studies have shown that continuous and prolonged monitoring with implantable devices increases detection of AF in populations that have high risk for stroke or have survived a stroke.^4^, ^5^, ^6^, ^7^, ^8^ Continuous long-term electrocardiographic (ECG) monitoring currently can be performed with implantable loop recorders, which are designed to overcome the limitations of intermittent monitoring, but the devices are expensive. However, implantable loop recorders are considered cost-effective in cryptogenic stroke patients.^9^ Noninvasive technologies for long-term monitoring could lead to cost savings in this population.

**Figure 1:**
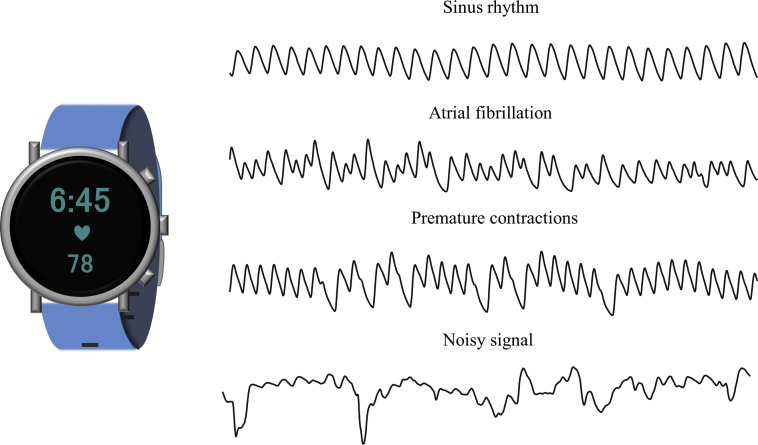
Smartwatch (*left*) and photoplethysmograph signals recorded during different cardiac rhythms and during a noisy signal (*right*).

Photoplethysmography (PPG) is a noninvasive, low-cost technology that can extract heart rate and rhythm information. PPG is an optical measurement modality that is used in many wrist-worn wearables, such as smartwatches, often accompanied by acceleration sensor measuring body movement. As a spot measurement, pulse and PPG techniques can be used with smartphone cameras, often in conjunction with the built-in flash. Smartphone camera-based PPG measurements have been proposed for discriminating AF.^10^, ^11^, ^12^ Subsequent studies have investigated AF detection using wrist-worn devices, with the aim of noninvasive long-term monitoring of AF in daily life. Figure 1 shows a smartwatch and PPG signals recorded during different cardiac rhythms.

The challenge with the new proposed solutions is that, as stated in the recent European Heart Rhythm Association position paper in relation to AF screening, “the role of consumer cardiac monitoring using wearables in combination with apps is completely undefined.”^13^ There have been two large-scale studies that monitored the general population in an ambulatory setting—the Apple Heart Study^14^ and the Huawei Heart Study.^15^ These studies were conducted by monitoring volunteers with wrist-worn PPG for several days and investigating whether wearing the device led to diagnosis of AF. From a technical perspective, the capabilities and limitations of PPG-based wrist-worn applications need to be well understood. Reviews in the literature have discussed mobile technologies, including PPG-based solutions, in terms of heart rate monitoring and AF detection^16^, ^17^, ^18^ but not from the perspective of wrist-worn PPG for long-term monitoring. This review focuses on devices that measure PPG at the wrist. The review summarizes the current knowledge on AF detection, discusses the potential and the knowledge gaps related to the technology in terms of long-term monitoring, and suggests directions for future research.

## Studies of wrist-worn devices for AF detection

This review summarizes studies on AF detection with wrist-worn devices that measured PPG and reported estimates of AF detection accuracy, published between September 2016 and September 2019. Aspects of these studies related to study populations, measurement setting and duration, methods for detection and PPG signal quality assessment, and detection performance and coverage are discussed. Details about the studies are given in the Online Appendix.

### Study populations

In the development and validation of new methods, such as detection of AF episodes using PPG, the size and diversity of the study population are important considerations in order to understand the generalizability of the proposed solutions. In the context of AF, diversity can be related to age, presence of comorbidities, use of medications, and different cardiac rhythms. When considering use of PPG technology for long-term monitoring, the person’s health status may have an impact on activity level. This, in turn, may influence the accuracy of the technology because PPG measurement is sensitive to movement artifacts.

The size of study populations varied from 11 to 1617 subjects. Most of the studies include fewer than 100 volunteers.^19^, ^20^, ^21^, ^22^, ^23^, ^24^, ^25^, ^26^, ^27^, ^28^, ^29^, ^30^, ^31^, ^32^, ^33^, ^34^ Six studies had a study population of more than 100 subjects^35^, ^36^, ^37^, ^38^, ^39^, ^40^; the largest monitored 1617 volunteers in an ambulatory setting.^36^

Rhythm characteristics in the study populations varied among studies. Some focused only on the distinction between AF and sinus rhythm, whereas others aimed to classify AF in the presence of other rhythms. Other rhythms included premature atrial contractions,^30^^,^^35^ premature ventricular contractions,^27^^,^^30^^,^^35^ ventricular arrhythmia,^31^ and atrial flutter, atrial tachycardia, and variable conduction.^27^ Corino et al^27^ and Fallet et al^31^ classified other rhythms as a separate class from AF and sinus rhythm. A number of studies did not specifically report what rhythms were included as non-AF or other rhythms.^20^^,^^21^^,^^23^, ^24^, ^25^^,^^28^^,^^36^ Shen et al^23^ reported the presence of 8 arrhythmias other than AF. Characteristics of AF vary among permanent, persistent, and paroxysmal forms because they evolve with time. Therefore, heart rate and rhythm complexity can be different depending on the type of AF.

In general, studies have been conducted in older populations of patients in whom AF is more prevalent. Most of the studies reported ages for both AF and non-AF groups, but some reported only the range or one average age. In most studies, the mean age of patients in the AF group was >70 years, with the lowest being 55.7 years. In the non-AF groups, the ages on average were slightly lower.

### Measurement setting and duration

The advantage of wrist-worn PPG devices is that they are noninvasive and comfortable to wear for long time periods in daily life. The extended monitoring time can be helpful when aiming to detect intermittent AF episodes, but ambulatory monitoring poses more challenges because of movement artifacts and compliance with device use.

In most of the studies, measurements were obtained in hospital or laboratory settings in which patients were in either a supine or sedentary position.^19^, ^20^, ^21^, ^22^^,^^26^, ^27^, ^28^^,^^31^, ^32^, ^33^, ^34^, ^35^, ^36^, ^37^, ^38^, ^39^, ^40^ The measurements were made before and after cardioversion,^26^^,^^32^^,^^34^, ^35^, ^36^ before or during catheter ablation or electrophysiological study,^19^^,^^31^^,^^39^ in the emergency care or cardiac ward,^40^ in postoperative patients,^22^^,^^33^ in admitted patients,^20^^,^^21^^,^^27^^,^^28^ and in laboratory settings.^34^^,^^37^^,^^38^ The recordings from these settings ranged from a few minutes to a few hours.

**Figure 2:**
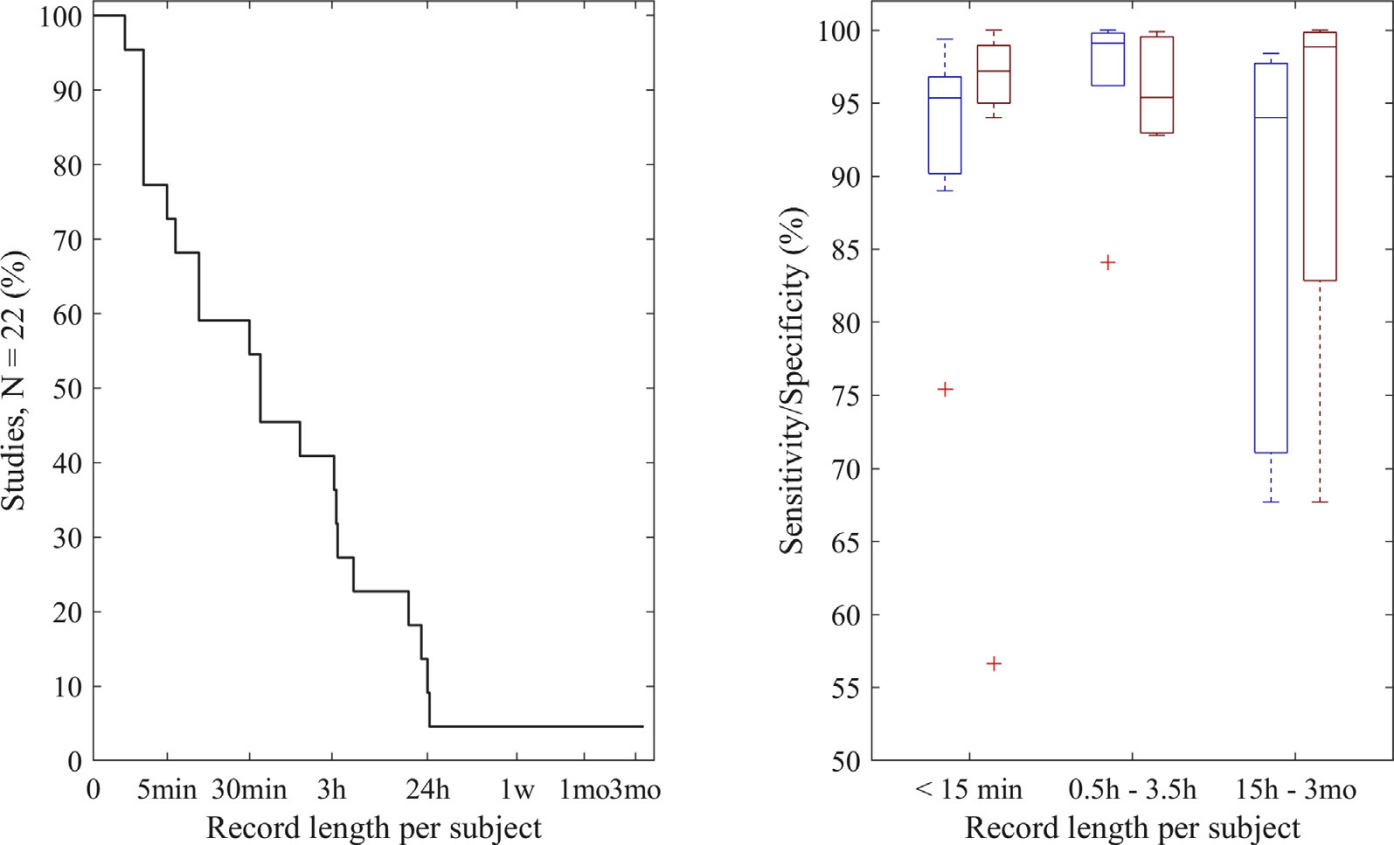
Percentage of studies monitoring on average at least a certain record length per subject (*left*) and sensitivity (*blue*) and specificity (*red*) of atrial fibrillation detection grouped by record length (*right*). Sensitivity was not reported in the study by Shashikumar et al^21^ and specificity was not reported in the study by Wasserlauf et al,^25^ and thus, those studies are not included.

Fewer studies have been performed in the ambulatory setting, with recordings in patients scheduled for Holter monitoring,^30^^,^^35^ patients in cardiac rehabilitation,^24^ patients with an implantable cardiac monitor,^25^ and an unspecified group of patients and healthy subjects.^23^^,^^36^ Figure 2 shows the percentage of studies that monitored subjects on average at least a certain record length (Figure 2, left), and the sensitivity and specificity of AF detection grouped by record length (Figure 2, right). Forty-five percent of the studies had recordings <30 minutes. For the studies that had both hospital and ambulatory cohorts, the record length based on the ambulatory cohort is included in the figure.

### Methods for AF detection and PPG data quality assessment

Various methods have been used for detecting AF episodes. The methods vary as to how easy they are to interpret and how easily their detection accuracy is affected when the signal is noisy. Excluding noisy data can improve detection accuracy, and for that reason automatic data quality assessment plays a role in automated AF detection.

A common method to detect the presence of AF from a PPG signal is assessing the irregularity of the rhythm from the interbeat interval (IBI) series. First, individual pulses (i.e., heart beats) are detected from the pulsatile PPG signal, and IBIs are calculated from the obtained beat times. Different features representing heart rate variability (e.g., pNN50,^22^^,^^27^ root mean square of successive differences^22^^,^^27^^,^^30^) or entropy (e.g., Shannon entropy,^30^ sample entropy,^20^^,^^27^^,^^30^ coefficient of sample entropy^30^^,^^40^) can be used to detect irregularity in the beat sequence. These features have been used by setting thresholds to determine whether AF is present,^30^^,^^39^^,^^40^ or they have been combined with machine learning to classify the rhythm.^19^^,^^20^^,^^22^^,^^27^ The IBI series also has been evaluated using a Markov model, which assigns to each interval a probability of being AF.^33^^,^^35^

In addition to time–domain analysis of IBIs, other features that could characterize the rhythm have been extracted from the signal. These include mainly features from the frequency domain, such as spectral entropy,^31^ spectral purity index,^31^ and wavelet power spectrum.^28^ Signal quality metrics have been combined with rhythm information, and the quality metrics have been derived from either the PPG signal itself or the accelerometer data characterizing movement that often is measured with PPG at the wrist.^20^ IBI features have been combined with this additional information using machine learning.^20^^,^^28^^,^^31^

Along with traditional machine learning methods, deep learning approaches have been proposed for AF detection from wrist-worn PPG.^21^^,^^23^^,^^25^^,^^28^^,^^29^^,^^32^^,^^36^ Shashikumar et al^21^^,^^28^ extracted first the time series and frequency–domain information as the input for their convolutional neural network. Two approaches have been based on extracted heart rate and activity data.^25^^,^^36^ Gotlibovych et al^32^ combined the PPG waveform and accelerometer data in their deep neural network consisting of convolutional and long-short term memory layers, whereas Aliamiri et al^29^ used this information first with one network to assess the quality of the PPG data and then classified the rhythm with a second network from the good-quality PPG data. Only one of the deep learning approaches was based only on the PPG waveform without additional quality assessments or other sensor information.^23^

PPG records are prone to artifacts due to movement or poor sensor contact. Therefore, robustness of the AF detection algorithm is an important aspect to consider when using PPG measured at the wrist. Poor-quality data can be discarded from the analysis, or the algorithm developed must be robust in the presence of noise. Many studies did not report assessment and exclusion of outliers or bad-quality data.^21^^,^^25^^,^^26^^,^^34^^,^^36^^,^^39^^,^^40^ Nemati et al^20^ and Shashikumar et al^28^ did not exclude data; instead they selected the best channel from 8 measured PPG channels for analysis based on signal quality metrics, which were derived from the raw PPG signal and accelerometer data. Deep learning approaches have been used to avoid discarding bad-quality data^23^^,^^32^ or to develop a separate signal quality assessment algorithm to select the data to be analyzed.^29^ Other methods to discard data as noisy include assessing motion level from the accelerometer data,^30^^,^^35^ using the standard deviation of the acceleration signals^31^ or deviation from the acceleration of gravity,^27^ combining signal-to-noise ratio information with accelerometer data,^37^ analyzing pulse morphology,^24^ and excluding outlier beats or IBIs.^19^^,^^33^ Harju et al^33^ selected the included beats based on whether they were correctly detected according to the ECG reference measurement.

### Detection performance and measurement coverage

For screening applications, it is important to be able to detect the presence of AF but not to cause false-positive detections. Falsely detected AF episodes are of concern and can harm the patient as well as burden the health care system. Movement artifacts, which occur frequently in daily life, can be an issue. Not making decisions about the presence of AF when the measured data are noisy improves detection accuracy but has a negative impact on measurement coverage. This, in turn, can lead to missing AF episodes.

Detection performance of the algorithms often is reported as sensitivity, specificity, and accuracy. However, all 3 metrics are not always reported, and some studies reported additional metrics. All but one of the studies that reported accuracy indicated values >90%.^19^, ^20^, ^21^^,^^24^^,^^26^, ^27^, ^28^, ^29^, ^30^, ^31^^,^^35^^,^^37^ Solosenko et al^24^ obtained accuracy of 87% when 89.2% of the 21-hour measurements were analyzed. However, when the quality requirement for the data to be analyzed was made stricter and 50% of the data were analyzed, the performance improved. Sensitivity improved from 72.0% to 97.2%, but improved accuracy was not reported.

Within all the studies that presented sensitivity and specificity,^19^^,^^20^^,^^22^^,^^24^^,^^27^^,^^28^^,^^30^, ^31^, ^32^, ^33^^,^^35^, ^36^, ^37^, ^38^, ^39^, ^40^ median sensitivity was 96.2% and specificity was 97.7%. Figure 2 shows sensitivities and specificities grouped by length of the recorded data per subject. Overall, the highest sensitivity reported was 100% when specificity was 93.1%.^34^ In the study in which 100% specificity was reported, sensitivity was 96% and 93% depending on the dataset.^35^ Gotlibovych et al^32^ reported close to 100% for both sensitivity and specificity (99.8% and 99.9%, respectively).

In addition to sensitivity and specificity, some studies reported the positive predictive value (PPV).^25^^,^^30^^,^^31^^,^^35^, ^36^, ^37^, ^38^, ^39^, ^40^ In hospital settings, the median PPV of the studies was 97.5% (82.0%–100%).^31^^,^^35^, ^36^, ^37^, ^38^, ^39^, ^40^ In ambulatory settings, false-positive findings were more common. Tison et al^36^ reported PPV of 90.9% when patients undergoing cardioversion were monitored but PPV of 7.9% in ambulatory settings. Wasserlauf et al^25^ reported PPV of 39.9% in a long-term study. In a study with 24-hour Holter monitoring and hard restrictions on not analyzing data affected by motion, PPV was 95.5%.^30^ Bonomi et al^35^ reported PPV of 100% in both cardioversion and Holter cohorts, with a slight drop in sensitivity from 96% to 93%. In their additional study population of 120 subjects free from arrhythmia^35^, 21% of the subjects had irregularities attributable to AF, and these episodes corresponded to <0.2% of total monitoring time.

In the studies that did not exclude any data, measurement coverage can be assumed to be 100%. With short measurements often obtained in hospital settings, measurement coverage has been 50%–95% after noise removal.^23^^,^^31^^,^^37^^,^^38^ In ambulatory settings, more disturbances to measurement are present and can influence the coverage. Shen et al^23^ reported final results of 100% coverage with measurements from 3 to 8 hours in ambulatory settings. In other ambulatory studies with measurement durations of approximately 24 hours, the coverage has been 25% to 58%,^30^ 47% to 48%,^35^ and 50% to 89.2%.^24^ The more selective the data quality assessment, the lower the coverage but the better the detection performance.^24^^,^^30^

## Discussion

### Clinical evidence and relevance

ECG currently is the gold standard for diagnosis of AF. PPG technologies as a tool for assisting with clinical decision-making in patients could be valuable for evaluating the need and requirements for ECG monitoring when AF is suspected. In addition, spot measurements of ECG could be made with wrist-worn devices that combine PPG and ECG,^16^^,^^20^ or with separate ECG devices connected to smartphones or tablets^41^ when continuous PPG-based measurements indicate rhythm irregularity.

An overview of studies investigating AF detection with wrist-worn PPG measurements has shown that excellent accuracy in general can be obtained. However, for long-term monitoring, study results should always consider the underlying circumstances, such as the diversity of the study population and the setting in which monitoring was performed. Most studies were conducted of hospitalized patients and in settings in which the study subjects were less active than during ambulatory monitoring. Fewer studies have been performed in ambulatory settings, in which movement artifacts are more likely to be present. Results of such studies vary more, especially the number of false-positive findings and PPV. Unreliable and bad-quality data can be discarded to improve detection accuracy, but this induces data loss, leading to a tradeoff between performance and measurement coverage. Figure 2 shows that in the group of studies with record length >15 hours, median specificity was very high, but the loss of data due to bad quality is not shown. No specificity value was included in the study by Wasserlauf et al,^25^ who reported only PPV (39.9%). Development of new techniques for robust detection methods are needed in order to obtain high accuracy and coverage in long-term ambulatory monitoring. In addition, studies assessing the impact of measurement coverage on diagnostic yield are needed.

The number of false-positive findings can be heavily influenced by other irregular rhythms. One study assessed the feasibility of AF screening with a smartphone camera.^42^ The study was based on a spot measurement of PPG initiated by the user from among 12,328 participants in the general population. Of the AF detections given by the algorithm, 23.7% were AF, but 34.2% were triggered by other irregularities, mainly frequent and irregular ectopic beats. Therefore, for development of AF detection methods, inclusion of other rhythm irregularities in the development data is important.

In many studies, subjects with AF were often known to have AF or otherwise to have a high probability for arrhythmia (e.g., hospitalized patients and patients undergoing cardiac procedures such as cardioversion or cardiac ablation). These populations may have different general health conditions, comorbidities, and medication use compared to the more general population targeted for screening.

The Apple Heart Study^14^ and the Huawei Heart Study^15^ aimed to study the feasibility of AF screening in large populations by monitoring participants with wrist-worn PPG for several days. Of the study populations, 0.5%^14^ and 0.2%^15^ were notified about irregular rhythms as an indication for them to contact a clinical expert. This seems reasonable considering the prevalence of AF. As a part of their inclusion criteria, both studies required participants to own both an adequate smartphone and smartwatch. The prevalence of AF increases with older age,^43^ however, smartwatches usually are owned by younger people.^44^ In the Huawei Heart Study, the mean age of the participants was 34.7 ± 11.5 years, with only <6% being older than 54 years.^15^ In the Apple Heart Study, 15.9% of participants were older than 54 years.^14^ Mass screening of the population that owns a consumer device, which generally is younger than the groups at risk, likely will not target those with undiagnosed AF and will increase the risk of false-positive findings in a presumably healthy population.

### Comparison of studies

The heterogeneity of study populations, measurement settings, methodological decisions such as the segment length to be classified, and metrics used to report the results makes objective comparison of methods and results difficult. This factor is important in the development of more accurate methods for detection of AF and possibly other arrhythmias. Currently, there is a lack of publicly available labeled datasets of wrist-worn PPG measurements in ambulatory settings that would make this comparison possible. The availability of a large dataset that is representative of the population and measurement conditions would enable a fair comparison of different solutions and allow more researchers to work on solutions. This, in turn, would accelerate technical developments and lead to more accurate solutions. For ECG analysis, such progress has been made possible because of the availability of various open source datasets and software, such as PhysioNet.^45^

Different studies reported their results with different metrics, and measurement coverage often was not explicitly reported. Considering the significance of false-positive findings, reporting PPV in addition to sensitivity and specificity is important. Coverage should be explicitly reported in order to determine whether data loss had an impact on diagnostic yield. In addition, subject characteristics, such as age and heart rhythms present during monitoring, may influence the performance of the detection method and therefore should be included.

### Future research and opportunities

The limited number of studies on AF detection by measuring wrist-worn PPG in ambulatory settings calls for more studies investigating how well the technology works in daily life, especially in detecting individual episodes and recording the duration of episodes. The optimal balance between measurement coverage and detection accuracy is not yet fully understood. If full measurement coverage with good accuracy can be obtained, continuous monitoring could enable better assessment of AF burden in addition to detection of AF. Moreover, more insight could be obtained on the daily patterns of AF episodes, whether they are related to triggers and certain symptoms, and whether medication is adequate for management of AF. For the latter, the accuracy of heart rate measurement during AF episodes is important in order to assess whether heart rate stays within desired limits. In the TACTIC-AF (Tailored Anticoagulation for Non-Continuous Atrial Fibrillation) study, intermittent anticoagulation guided by continuous monitoring of AF burden with pacemakers and implantable cardioverter-defibrillators yielded promising results in patients with rare AF episodes and low-to-moderate stroke risk.^46^ More research on the topic is warranted, but these results on guided medication intake are another indication that unobtrusive continuous AF monitoring could have value beyond screening if the technical challenges related to measurement coverage can be overcome.

Studies similar to the Apple Heart Study and the Huawei Heart Study targeting populations at risk are currently lacking. The results and the accuracy of the solutions may change if most of the monitored individuals have higher risk for AF but possibly other arrhythmias and comorbidities as well. There is a need for randomized trials to assess whether detecting AF with wrist-worn devices in daily life leads to safe and effective treatment for reducing strokes. The relationship between stroke risk and duration of device-detected AF has been studied with implantable devices, with varying results.^47^^,^^48^ Therefore, insight on stroke risk related to AF detected with wrist-worn wearables is important in order to determine the value of the technology both for maintaining people healthy and improving quality of life and for saving costs and improving the health care system.

## Conclusion

Studies investigating AF detection with wrist-worn PPG measurements show promising results. Many studies have been conducted with short measurements and with hospitalized patient groups; studies in ambulatory settings are fewer. In an ambulatory setting, the challenge is balancing accuracy and measurement coverage when aiming for continuous monitoring. With intermittent monitoring, large-scale studies have shown that mass screening of people who own consumer devices, who in large part are younger than the populations at risk, does not cause an excessive number of false-positive findings but does lead to diagnosis of AF at follow-up. More studies of long-term monitoring among at-risk populations are warranted to determine how well the technology works in these circumstances. Technical developments should aim to improve measurement coverage while maintaining high detection accuracy. Publicly available datasets would accelerate such developments. Accurate continuous long-term monitoring could open new opportunities to move beyond AF screening toward disease management.

## Funding Sources

This work was carried out within the framework of the Eindhoven MedTech Innovation Center (e/MTIC) as a collaboration between Eindhoven University of Technology, Catharina Hospital Eindhoven, and Philips Research, and partly supported by ITEA3 project, 15032, eWatch.

## Disclosures

L.M. Eerikäinen is a guest researcher at Philips Research and dr. A.G. Bonomi is an employee of Philips Research. All other authors have no conflicts relevant to the contents of this paper to disclose.
