# Supplementary material for: Atrial fibrillation monitoring with wrist-worn photoplethysmography-based wearables: State-of-the-art review
Source: Cardiovasc Digit Health J. 2020 Aug 26;1(1):45–51. doi: 10.1016/j.cvdhj.2020.03.001 (PMC8890076; doi:10.1016/j.cvdhj.2020.03.001)
Supplement: Online Appendix [file mmc1.docx]

Appendix

Table 1 Studies with wrist-worn devices.

| **Author** | **Number of subjects** | **Rhythm types** | **Age (years)** | **Device** | **Duration of recording** | **Detected episode duration** | **Measurement setting** | **Reference data** | **Detection performance** | **Measure-ment coverage** |
| --- | --- | --- | --- | --- | --- | --- | --- | --- | --- | --- |
| Lemay et al.^19^ | 20 | AF and SR | Not reported | Proprietary device | 2213 10 s-epochs^¶^, one epoch per detected beat | Single beat | Hospital before catheter ablation | 12-lead ECG annotated by epoch | Sens: 99.38%  Spec: 56.64%  Acc: 93.76% | Not explicitly reported |
| Nemati et al.^20^ | 46 (36+10) | AF and other rhythms | 18-89 | Samsung Simband | 3.5-8.5 min per subject | Each record | Hospital | Single-lead ECG | Sens: 97%  Spec: 94%  Acc: 95% | Not explicitly reported |
| Corino et al.^27^ | 70 | AF, other arrhythmia, and SR | AF: 76 ± 9 (58-89)  SR: 40 ± 17 (27-75)  Other: 65 ± 15 (48-92) | Empatica E4 | 10 min per subject | 2 min | Hospital | Not reported | ^†^Sens: 75.4%  ^†^Spec: 96.3% | 20% (the best 2 min segment selected) |
| Shashikumar et al.^28^ | 98 | AF and other rhythms | 18-89 | Samsung Simband | Approx. 5 min per subject | 30 s | Hospital | Single-lead ECG | Sens: 89%  Spec: 96%  Acc: 91.8% | Not explicitly reported |
| Aliamiri et al.^29^ | 19 | AF and SR | Not reported | Samsung gear device | 1443 30 s epochs | 30 s | Not reported | ECG | Acc: 98.18% | 50% |
| Bonomi et al.^35^ | 20+40 (+120)^‡^ | AF and other rhythms | 73.1 ± 11.6 (45-87) and 67.4 ± 12.1 (34-87) | Data logger with Philips C3M module | 1.9–2.8 h and 21.9–39.2 h per subject | 1 min | Hospital ECV and daily life | Single-lead ECG and 12-lead ECG | Sens: 96% and 93%  Spec: 100% and 100%  Acc: 98% and 97% | 47% and 48% |
| Eerikäinen et al.^30§^ | 30 | AF and other rhythms | AF: 69 ± 11 (43-79)  Other: 67 ± 13 (34-87) | Data logger with Philips C3M module | 24 h per subject | 2 min | Daily life | 12-lead ECG | Sens: 98.4%  Spec: 98.0%  Acc: 98.1% | 25% |
| Fallet et al.^31^ | 17 | AF, ventricular arrhythmias, and SR | 57 ± 13 | Proprietary device | 3056 10 s-epochs | 10 s | Hospital during ablation procedure | 12-lead ECG | ^†^Sens: 96.2%  ^†^Spec: 92.8%  ^†^Acc: 95.0% | 71% |
| Gotlibovych et al.^32^ | 53 | AF and SR | 37-85 | Prototype fitness tracker device | 180 h data (36 h AF)^¶^ | 0.8 s | Hospital ECV and during sleep outside hospital | ECG | Sens: 99.8%  Spec: 99.9% | 100% |
| Harju et al.^33^ | 30 | AF and SR | AF: 74.8 ± 8.3  SR: 67.5 ± 10.7 | PulseOn optical heart rate monitor | 1-2 h per subject | 1 min | Hospital during post-operative care | ECG | Sens: 99.0%  Spec: 92.96% | Not explicitly reported |
| Hochstadt et al.^34^ | 20 | AF and SR | 74.1 ± 8.7 | CardiacSense | 30 min per subject | 30-250 beats | Hospital or laboratory | ECG | Sens: 100%  Spec: 93.1% | Not explicitly reported |
| Shashikumar et al.^21^ | 97 | AF and other rhythms | 18-89 | Samsung Simband | 5-10 min per subject | 30 s | Hospital | ECG | Spec: 100%  Acc: 95% | Not explicitly reported |
| Tison et al.^36^ | 51+1617 | AF and other rhythms | 66.1 ± 10.7 and  AF: 55.7 ± 14.2  Other: 41.4 ± 11.9 | Apple Watch | 40 min and 18.5 million 5 s measure-ments^¶^ | 5 s | Hospital CV and daily life | 12-lead ECG and self-reported persistent AF | Sens: 98.0% and 67.7%  Spec: 90.2% and 67.6% | Not reported |
| Yousefi et al.^22^ | 30 | AF and SR | AF: 74.8 ± 8.3  SR: 67.5 ± 10.7 | PulseOn optical heart rate monitor | 1.5 h | 30 IBIs | Hospital during post-operative care | ECG | Sens: 99.2% ± 1.3%  Spec: 99.54% ± 0.64% | 73.3% |
| Dörr et al.^37^ | 672 | AF and SR | AF: 77.4 ± 9.1  SR: 75.6 ± 9.8 | Samsung Gear Fit 2 | 5 min | 1 min | University center | iECG | Sens: 93.7%  Spec: 98.2%  Acc: 96.1% | 78% |
| Fan et al.^38^ | 108 | AF and SR | AF: 66.56 ± 13.17  SR: 58 ± 14.78 | Huawei Band 2 | 3 min | 1 min | Hospital | 12-lead ECG | Sens: 95.36%  Spec: 99.70% | 95% |
| Kashiwa et al.^39^ | 20+116 | AF and other rhythms | 62.7 ± 10.9 | Pulse wave monitor, Seiko Epson | 368 h^¶^ | 6 min | Hospital during electro-physiological study | 2-channel ECG | Sens: 84.1%  Spec: 97.7% | 100% |
| Shen et al.^23^ | 82 | AF and other rhythms | Not reported | Not reported | 8 h and 3 h | 30 s | Daily life | ECG patch | Only AUC reported (94.8%) | 100% |
| Sološenko et al.^24^ | 34 | AF and other rhythms | AF: 72.9 ± 8.9  Other rhythms: 67.5 ± 10 | Wrist-worn device developed at Kaunas University of Technology | 21 h | One pulse | Cardiac rehabilitation | Single-lead ECG | Sens: 72.2%  Spec: 99.7%  Acc: 87.0% | 89.2% |
| Väliaho et al.^40^ | 213 | AF and SR | AF: 72.0 ± 14.3  SR: 54.5 ± 18.6 | Empatica E4 | 5 min | 2 min | Hospital, emergency care or cardiac ward | 3-lead ECG | Sens: 96.2%  Spec: 98.1% | Not reported |
| Wasserlauf et al.^25^ | 24 | AF and other rhythms | 72.1 ± 7.2 | Apple Watch | 110 ± 35.7 days | 1 h | Daily life | ICM | Sens: 97.5% | Not reported |
| Yang et al.^26^ | 11 | AF and SR | 63 ± 12 | Prototype device | 0.75-12 h | 10 s | Hospital ECV | ECG | Acc: 92.71% | Not reported |

Acc = accuracy, AF = atrial fibrillation, ECV = elective cardioversion, ICM = insertable cardiac monitor, IBI = inter-beat interval, Sens = sensitivity, SR = sinus rhythm, Spec = specificity

^†^Performance reported only for AF detection

‡Details about these subjects are not included in the table

§Only the best performance reported

¶Total dataset
